# Patient Characteristics, Treatment, and Survival in Pleuropulmonary Blastoma: An Analysis from the National Cancer Database

**DOI:** 10.3390/children11040424

**Published:** 2024-04-02

**Authors:** Charbel Chidiac, Shelby R. Sferra, Shaun M. Kunisaki, Daniel S. Rhee

**Affiliations:** 1 Department of Surgery, Johns Hopkins University School of Medicine, Baltimore, MD 21287, USA; cchidia2@jh.edu (C.C.); drhee1@jhmi.edu (D.S.R.); 2 Department of Surgery, Columbia University Vagelos College of Physicians and Surgeons, New York, NY 10032, USA

**Keywords:** pleuropulmonary blastoma, chemotherapy, radiation, survival

## Abstract

Pleuropulmonary blastoma (PPB) is a rare childhood tumor originating from the lung or pleura, typically treated with surgery, chemotherapy (CTx), and/or radiation therapy (RTx). This study aimed to assess patient and tumor features, treatment methods, and survival rates in PPB. We retrospectively analyzed PPB patients under 18 from 2004 to 2019, using the National Cancer Database (NCDB). We assessed 5-year overall survival (OS) rates based on disease presentation and treatment regimens, using Kaplan–Meier curves and Cox proportional regression. Among 144 cases identified, 45.9% were female, with a median age of 2 years (interquartile range 1–3). In all, 62.5% of tumors originated from the lung, and 10.4% from the pleura. Moreover, 6.9% were bilateral, and the median tumor size was 8.9 cm, with 4.2% presenting with metastases. The 5-year OS rate was 79.6%, with no significant change over time (2004–2009, 77.6%; 2010–2014, 90.8%; 2015–2019, OS 75.3%; *p* = 0.08). In all, 5.6% received CTx alone, 31.3% surgery alone, 45.8% surgery/CTx, and 17.4% surgery/CTx/RTx. The OS rates were comparable between the surgery/CTx/RTx (80.0%) and surgery/CTx (76.5%) groups (adjusted Hazard Ratio, 1.72; 95% CI, 0.57–5.19; *p* = 0.34). Therefore, due to the unchanged survival rates over time, further prospective multicenter studies are needed to determine the optimal multimodal treatment regimens for this rare pediatric tumor.

## 1. Introduction

Pleuropulmonary blastoma (PPB) is a rare and aggressive, dysembryonic, malignant tumor of early childhood that originates from the lung, pleura, or both [1,2]. It was initially described in 1988, and since then, over 500 cases have been reported in the literature [3,4]. PPB follows a progression pathway characterized by three subtypes: type I (cystic), type II (cystic/solid), and type III (completely solid) [4]. Furthermore, a germline mutation in DICER1 has been associated with 65 to 70% of PPB cases [5,6,7].

Given its rarity and diverse clinical manifestations, PPB has been associated with variable survival rates, ranging from 49% to 91% based on single-center reports and voluntary cancer registry studies [1,4,8,9]. The treatment of PPB typically involves surgical resection of the tumor, while the use of adjuvant therapies such as chemotherapy (CTx) and/or radiation therapy (RTx) depends on multiple factors, including PPB subtype, extent of initial resection, presence of metastases at diagnosis, and disease recurrence [10,11]. This study aimed to provide a contemporary analysis of patient and tumor characteristics, treatment regimens, and survival outcomes, using a national hospital-based cancer database.

## 2. Materials and Methods

### 2.1. Data Source and Study Population

Data were extracted from the National Cancer Database (NCDB) Participant User File. The NCDB is a joint project of the Commission on Cancer of the American College of Surgeons and the American Cancer Society. Established in 1989, this database encompasses almost 72% of all newly diagnosed cases of cancer across the United States collected from a cancer program that is accredited by the American College of Surgeons Commission on Cancer (CoC) [12]. The 2019 PUF Release, which was used for this study, includes data for patients diagnosed in 2004 through 2019. Histological diagnoses are confirmed by pathologists at each CoC institution, following the International Classification of Disease for Oncology 3rd Edition (ICD-O-3) guidelines and before submission to the NCDB. Facility-specific information was de-identified in accordance with the requirements of the Health Insurance Portability and Accountability Act (HIPAA). The American College of Surgeons and the Commission on Cancer have not verified and are not responsible for the analytic or statistical methodology employed, or the conclusions drawn from these data by the investigator.

This study was limited to patients with less than 18 years of age with a histologically confirmed diagnosis of PPB between 2004 and 2019. The dataset was queried using tumor histology codes from the International Classification of Disease for Oncology 3rd Edition (ICD-O-3) codes 8972 and 8973 [13].

### 2.2. Variables

Demographic variables included age at diagnosis, sex, race, and ethnicity. Tumor characteristics, such as tumor primary site, laterality, size, presence of metastases at diagnosis, and surgical margins, were recorded. Four different regimens were used in the treatment of patients in the cohort; these include definitive surgery of the primary tumor alone (excluding patients undergoing surgery for metastasis excision without primary tumor excision or those who had only a tumor biopsy), chemotherapy alone, surgery with either adjuvant or neoadjuvant chemotherapy, and surgery followed by combined CTx and RTx.

The primary outcome was the 5-year overall survival (OS) defined as the number of months from the patient’s initial diagnosis with PPB to either death within five years or day of last contact. The NCDB does not collect cancer-specific survival, and the cause of death is not specified. To explore potential improvements in survival over time, patients were further stratified into three groups, each spanning a 5-to-6-year interval: 2004–2009, 2010–2014, and 2015–2019. Further subdivision or categorization by each individual year was not feasible due to the low number of patients, which made it challenging to ensure statistical robustness and meaningful analysis.

### 2.3. Statistical Analysis

Continuous variables were reported as median with interquartile range (IQR) after confirming non-normal distribution using the Shapiro–Wilk test of normality, whereas categorical variables were reported as frequencies with corresponding percentages. Analysis of Variance (ANOVA) or the Kruskal–Wallis rank sum test was used to compare continuous variables, while Chi-square or Fisher’s exact tests were used to compare categorical variables. In cases where the *p*-values were significant, a post hoc analysis was conducted to examine the pairwise differences between the groups (File S2).

To assess overall survival and survival differences among various treatment regimens (surgery alone, CTx alone, surgery/CTx, and surgery/CTx/RTx) and years of diagnosis, a survival analysis was conducted using Kaplan–Meier methods. Additionally, a univariate Cox proportional hazard analysis was performed to evaluate the impact of demographic and tumor factors on mortality, including age, sex, race, ethnicity, laterality, tumor size, metastasis at diagnosis, and surgical margins (Table S1). To construct a robust multivariate Cox proportional hazard model, covariates with a *p*-value < 0.20 from the univariate analysis or those identified as potential confounders through clinical judgment (such as surgical margins) were considered. The final model incorporated treatment modality, tumor size (represented as a binary categorical variable using a literature-derived cutoff of 5 cm [14,15]), ethnicity, metastasis at diagnosis, and surgical margins as significant covariates. A *p*-value of less than 0.05 was considered statistically significant. All statistical analyses were performed using R Statistical software (version 4.2.2).

## 3. Results

A total of 144 children with a primary diagnosis of PPB were identified. The median age of the cohort was 2 years (interquartile range (IQR), 1–3), with only 15 (10.4%) patients being older than 5 years. Sixty-six (45.9%) patients were female. There were 120 (83.4%) White, 17 (11.8%) Black, and 7 (15.3%) Hispanic patients (Table 1).

Most tumors (*n* = 120, 83.4%) primarily originated from the lung or bronchus, with no preference between the upper or lower lobes (28.5% and 24.3%, respectively; Table 2). Seventy-one (49.3%) patients had a unilateral right-sided tumor, and 62 (43.1%) had a left-sided one. One patient (0.7%) had bilateral disease. The median tumor size was 8.9 cm in diameter, but 43 (29.9%) cases had missing data on tumor size. Metastases were present at diagnosis in six patients (4.2%), with two to the bone, one to the brain, one to the lung, and two to other sites besides the liver and distant lymph nodes. Eight patients (5.5%) were treated with chemotherapy alone, and the remainder (94.4%) had a surgical resection of the tumor. Surgery was the only treatment in 45 patients (31.3%), while 66 patients (45.8%) received additional CTx (2 neoadjuvant and 64 adjuvant), and 25 patients (17.3%) received adjuvant CTx and RTx. Among patients with metastases, only one out of six underwent surgery for metastasis excision (Table S2).

Compared to patients treated with surgery alone, patients who received CTx alone showed a trend towards a higher median age (CTx alone = 3 years vs. surgery alone = 0.5 years, *p* = 0.08), more tumors in unspecified location (50% vs. 6.7%, *p* = 0.01) or thorax/mediastinum (25% vs. 4.4%, *p* = 0.07), a larger median tumor size (11.9 cm vs. 4.5 cm, *p* = 0.2), and more metastases at diagnosis (12.5% vs. 0%, *p* = 0.06). Compared to those treated with surgery/CTx, patients treated with surgery/CTx/RTx had no difference in median age (surgery/CT/RTx = 3 years vs. surgery/CTx = 2 years), tumor size (9.5 cm vs. 9.5 cm) and tumor location; however, they had more metastasis at diagnosis (12.0% vs. 3.0%, *p* = 0.04) and more positive surgical margins (40.0% vs. 25.8%, *p* = 0.001) (Table 2).

**Figure 1:**
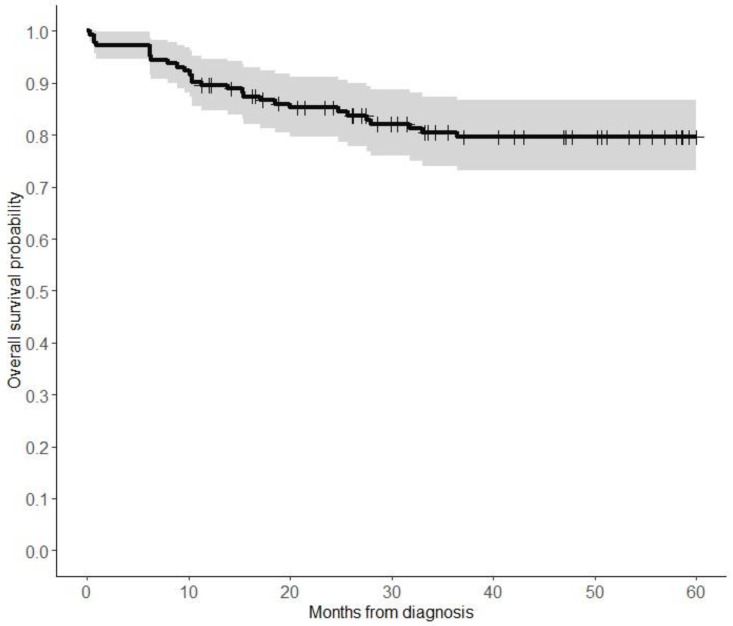
Overall survival of all patients with pleuropulmonary blastoma Kaplan–Meier survival curve for patients with pleuropulmonary blastoma, showing survival probability. Censoring events are marked by vertical ticks. Shaded area represents 95% confidence interval.

**Figure 2:**
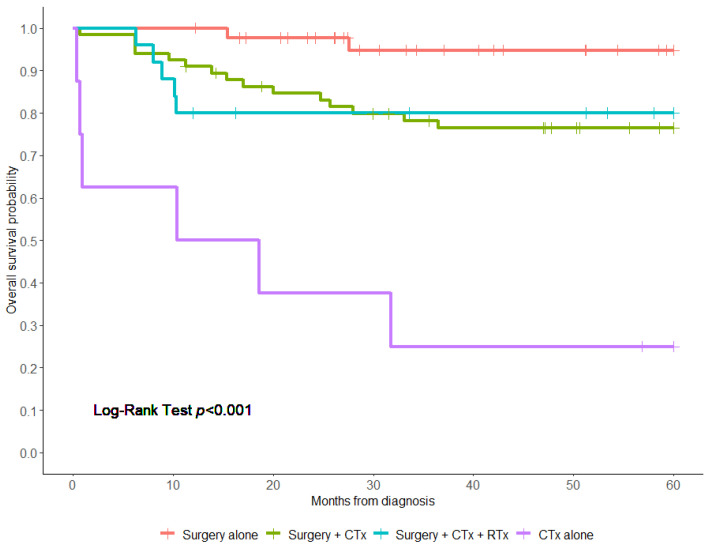
Overall survival of patients with pleuropulmonary blastoma based on their treatment regimen: surgery alone, surgery and chemotherapy (Surgery/CTx), surgery, chemotherapy and radiation therapy (Surgery/CTx/RTx), and CTx alone. Censoring events are marked by vertical ticks. Log-rank test was performed to compare the 4 groups.

**Figure 3:**
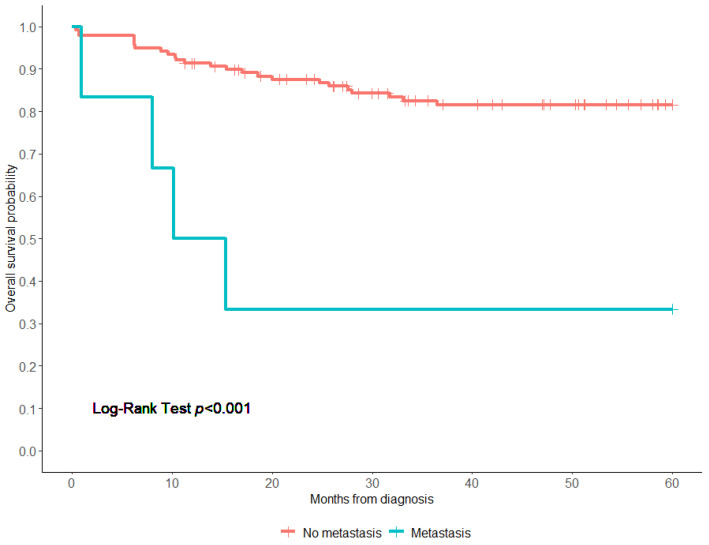
Overall survival of patients with pleuropulmonary blastoma based on presence of metastasis at diagnosis. Censoring events are marked by vertical ticks. Log-rank test was performed to compare the 2 groups.

**Figure 4:**
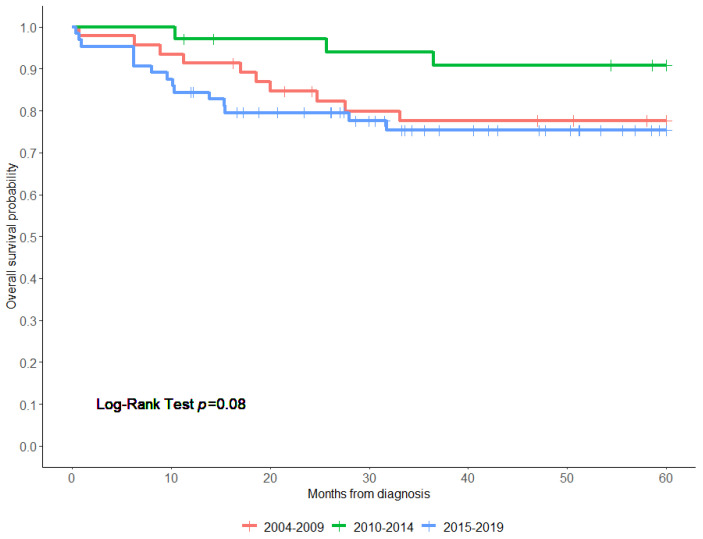
Overall survival of patients with pleuropulmonary blastoma based on the study period: 2004–2009, 2010–2014, and 2015–2019. Censoring events are marked by vertical ticks. Log-rank test with a degree of freedom of 2 was performed to compare the 3 groups.

The 5-year OS rate of all patients was 79.6% (95% confidence interval (CI), 75–87%) (Figure 1). Patients treated with surgery alone had a significantly higher 5-year OS than those who had surgery/CTx or surgery/CTx/RTx (94.8% vs. 76.5% vs. 80.0%, respectively, log-rank test; *p* < 0.0001). Patients who received chemotherapy alone had the worst OS (25.0%) (Figure 2). Notably, patients who presented with metastasis had a worse 5-year overall survival compared to those who did not have metastasis (33.3% vs. 82.1%, *p* < 0.001) (Figure 3). Additionally, there were no significant differences in the 5-year OS of patients based on study period (2004–2009 OS, 77.6%; 2010–2014 OS, 90.8%; and 2015–2019 OS, 75.3% (*p* = 0.08)) (Figure 4 and Figure S1). On the multivariate analysis, the presence of metastasis at diagnosis was associated with a worse survival (adjusted Hazard Ratio (aHR), 9.14; 95% CI, 2.22 -37.7; *p* < 0.002) (Table 3).

## 4. Discussion

In this PPB study of 144 patients utilizing the NCDB, we confirmed that PPB primarily affects infants and young children given a median age of diagnosis of 2 years old. Second, we found that bilateral thoracic disease and metastatic spread at diagnosis are relatively uncommon but are associated with a worse prognosis. Third, the 5-year OS was 79.6%, although no apparent survival improvement was observed over the 16-year period. Fourth, surgical resection is a critical component in the effective management of PPB given its association with higher survival rates when compared to CTx alone. Finally, adjuvant RTx was not associated with improved survival when compared to those receiving surgery and CTx. Taken together, these contemporary data add to the existing literature on our understanding of this rare pediatric tumor [4,14,16].

Our study, spanning from 2004 to 2019, reveals a median age at diagnosis of 2 years, aligning with the findings from other studies indicating a median age of 30–37 months [8]. We noted a metastatic rate of 4.2% and an equal male-to-female ratio of 1:1, similarly to the 4.9% metastatic rate reported by González et al. in their analysis of 143 cases [17]. In contrast, Messinger et al. examined 350 cases from the PPB registry across 42 countries from 1988 to 2012, revealing a higher metastatic rate of 9% [4]. Both studies reported an equal male-to-female ratio. Initially described in 1988, the prognosis for patients with PPB was thought to be poor [17]. Previous studies reported survival rates of approximately 50–70% [3,4,8,15], significantly lower than our reported 5-year overall survival of 79%. Earlier diagnosis, advancements in surgical techniques, the development of novel chemotherapeutic agents, and a deeper understanding of the disease itself are likely factors contributing to improved survival rates in PPB [18]. Extrapulmonary involvement [8], the presence of metastasis at diagnosis [4], the degree of surgical resection [8,9], and tumor size [15] are some of the factors that were shown to affect the prognosis of patients with PPB. In our cohort, the presence of metastasis at the time of diagnosis was identified as the only factor associated with a worse prognosis.

Type I PPB generally has a favorable prognosis, with a reported survival rate of 91% [1,4,14]. Type I PPB is purely cystic. Its treatment typically involves surgery alone. An indication for chemotherapy would be if the primary tumor is not completely resected or there was intraoperative tumor spill (cyst rupture) during the operation. Single-institution experiences have shown that gross total resection is highly recommended for cure, and that it is achievable with minimal surgical morbidity and a good prognosis [11,18]. Type I PPB is clinically and radiologically indistinguishable from macrocystic congenital pulmonary airway malformation (CPAM), a benign cystic lung mass most commonly detected in utero [19,20,21]. On the other hand, type II and III PPB usually receive adjuvant chemotherapy [16,22,23] and have lower survival rates, 59% and 37%, respectively. Currently, there are no standard CTx regimens designed specifically for PPB. Vincristine, actinomycin-D, doxorubicin, cisplatin, cyclophosphamide, and irinotecan are the common agents used [3,16,24,25]. It is important to note that the higher survival rate observed in our study may have been driven by a high proportion of PPB type I cases, assuming that these were the patients treated with only surgery. In contrast, data from the International PPB Registry show a relatively equal distribution of the different PPB subtypes [17,23].

Patients who underwent a combined treatment approach involving CTx and surgery appeared to exhibit specific clinical characteristics, including advanced age, larger tumor size, positive surgical margins, and the presence of metastasis, compared to those that underwent surgery only. Conversely, patients receiving RTx in conjunction with CTx displayed similar baseline attributes to those receiving CTx alone, albeit with a slightly elevated incidence of positive surgical margins and metastasis. Surprisingly, our findings indicated that the incorporation of CTx and RTx into the treatment regimen did not yield superior outcomes compared to CTx alone in the post-surgical management of PPB patients. We made concerted efforts to mitigate potential confounding factors, such as patient age, extent of tumor resection, tumor dimensions, and metastasis, at the time of diagnosis. Nevertheless, it is essential to underscore that the absence of recorded data pertaining to recurrence patterns and progression-free survival in the utilized database restricts the depth of our insights. [26].

Considering the young age of PPB patients, it is imperative to exercise caution and prudence in the utilization of radiation therapy. Radiation should be contemplated solely in specific and critical cases after a comprehensive assessment of associated risks and benefits. One compelling reason to incorporate RTx is its potential to enhance local tumor control. In instances where tumors are substantial in size or surgical margins are positive, RTx can effectively target residual cancer cells, thereby diminishing the likelihood of local recurrence [27]. Nevertheless, the exposure to radiation, especially at elevated doses, heightens the risks of short-term adverse effects, future malignancies, and the development of pulmonary fibrosis [28].

Several factors may contribute to the absence of significant differences in outcomes between the groups receiving neoadjuvant or adjuvant CTx alone and those receiving a combination of CTx and RTx: Firstly, the study’s sample size warrants consideration. It is essential to assess whether the study featured a sufficiently large sample size to detect statistically significant differences. Secondly, patients undergoing RTx may have presented with more challenging tumor characteristics, such as larger tumor sizes or positive surgical margins. If RTx was predominantly administered to patients with more advanced disease, this differential patient selection could account for the lack of discernible differences in outcomes [29]. Lastly, it is important to acknowledge the potential influence of unaccounted factors. The study may not have comprehensively considered all variables impacting outcomes, including genetic factors, resectability of the primary tumor, variations in specific chemotherapy regimens, or differences in individual patient responses to treatment [30]. These unaccounted factors could have masked the potential benefits of adding RTx to the treatment regimen. Additionally, the database does not provide information on the specific type of surgery that was performed and does not include the Current Procedural Terminology (CPT) codes of the operation. Furthermore, the specific chemotherapy agents or regimens, along with their dosing and number of cycles, were not available. Finally, the absence of data on CPAM-related factors, such as previous CPAM, prenatal diagnosis, or incidental diagnosis, represents a notable limitation.

The NCDB is not without its limitations. Some large independent children’s hospitals did not participate in the NCDB [31], and given its retrospective design, the database has missing data and lacks specific covariates. For example, the most crucial factor in predicting the outcome of PPB is its subtype, but unfortunately, we were unable to determine the subtypes in our study. We were also unable to obtain data on the presence of DICER1 gene mutations. DICER1 gene testing has been suggested as the initial step in the management of PPB, as approximately 70% of cases showed a mutation in this gene [4,5]. Mutations in DICER1 have been associated with the progression of tumors from mild to more severe subtypes [17]. Nevertheless, the NCDB offers broader generalizability of our findings compared to single-institution studies, statewide databases, and voluntary registries, as it encompasses approximately 72% of cancer patients treated throughout the US. While the NSQIP-P database focuses solely on patients who underwent surgery, potentially excluding non-surgical cases [32], and the SEER database covers around 34.6% of the US population (https://seer.cancer.gov (accessed on 25 March 2024)), the PPB/DICER1 Registry has been extensively utilized for PPB research [4,23,26]. Nonetheless, the NCDB presents a novel opportunity, as it has not been previously explored for PPB studies, potentially offering new insights into the disease.

In conclusion, in our study utilizing a national cancer database, most PPB cases present as unilateral disease in infants and toddlers. Although the 5-year OS was 79.6%, survival rates appear to be stagnant, without improvement over time. Further prospective multicenter studies are necessary to provide more definitive recommendations and to enhance our understanding of this rare disease.

## Figures and Tables

**Table 1:** Demographics of patients undergoing treatment for pleuropulmonary blastoma.

	Total *n* = 144	Surgery *n* = 45	Surgery/CTx *n* = 66	Surgery/CTx/RTx *n* = 25	CTx *n* = 8	*p*-Value
Age, median years (IQR)	2 (1–3)	0 (0–2)	2 (1–3)	3 (2–4)	3 (2–3.25)	0.08
Sex, *n* (%)						0.55
Male	78 (54.2)	24 (53.3)	33 (50.0)	15 (60.0)	6 (75.0)	
Female	66 (45.9)	21 (46.7)	33 (50.0)	10 (40.0)	2 (25.0)	
Race, *n* (%)						0.20
White	120 (83.4)	40 (88.9)	54 (81.8)	20 (80.0)	6 (75.0)	
Black	17 (11.8)	4 (8.9)	10 (15.2)	3 (12.0)	0 (0.0)	
Other	7 (4.9)	1 (2.2)	2 (3.0)	2 (8.0)	2 (25.0)	
Ethnicity, *n* (%)						0.53
Hispanic	22 (15.3)	8 (17.8)	10 (15.2)	2 (8.0)	2 (25.0)	
Non-Hispanic	122 (84.7)	37 (82.2)	56 (84.9)	23 (92.0)	6 (75.0)	
Year at diagnosis, *n* (%)						**0.008**
2004–2009	46 (31.9)	7 (15.6)	24 (36.4)	13 (52.0)	2 (25.0)	
2010–2014	34 (23.6)	9 (20.0)	20 (30.3)	3 (12.0)	2 (25.0)	
2015–2019	64 (44.4)	29 (64.4)	22 (33.3)	9 (36.0)	4 (50.0)	

**CTx**, chemotherapy; **IQR**, interquartile range; **RTx**, radiation therapy; **SD**, standard deviation. *p*-values in bold indicate statistical significance, *p <* 0.05

**Table 2:** Tumor characteristics of patients undergoing treatment for pleuropulmonary blastoma.

	Total *n* = 144 (%)	Surgery *n* = 45 (%)	Surgery/CTx *n* = 66 (%)	Surgery/CTx/RTx *n* = 25 (%)	CTx *n* = 8 (%)	*p*-Value
Location						
Upper lobe	41 (28.5)	22 (48.9)	13 (19.7)	6 (24.0)	0 (0.0)	**0.002**
Middle lobe	14 (9.7)	4 (8.9)	8 (12.1)	2 (8.0)	0 (0.0)	0.70
Lower lobe	35 (24.3)	11 (24.4)	17 (25.8)	6 (24.0)	1 (12.5)	0.87
Pleura	15 (10.4)	3 (6.7)	7 (10.6)	4 (16.0)	1 (12.5)	0.67
Bronchus/lung Unspecified	30 (20.8)	3 (6.7)	16 (24.2)	7 (28.0)	4 (50.0)	**0.01**
Thorax/mediastinum	9 (6.3)	2 (4.4)	5 (7.6)	0 (0.0)	2 (25.0)	0.07
Laterality						0.26
Right	71 (49.3)	24 (53.3)	33 (50.0)	10 (40.0)	4 (50.0)	
Left	62 (43.1)	19 (42.2)	26 (39.4)	15 (60.0)	2 (25.0)	
Both	1 (0.7)	0 (0.0)	1 (1.5)	0 (0.0)	0 (0.0)	
Unknown	10 (6.9)	2 (4.4)	6 (9.1)	0 (0.0)	2 (25.0)	
Tumor size (cm), median [IQR]	8.9 [5.0–1.1]	4.5 [19.5–7.6]	9.5 [6.6–11.0]	9.5 [9.0–11.5]	11.9 [11.1–13.0]	0.2
≤5 cm	27 (18.8)	19 (42.2)	7 (10.6)	1 (4.0)	0 (0.0)	0.13
>5 cm	74 (51.4)	17 (37.8)	36 (54.6)	16 (64.0)	5 (62.5)	
Unknown	43 (29.8)	9 (20.0)	23 (34.9)	8 (32.0)	3 (37.5)	
Surgical margins						**0.001**
Positive	29 (20.1)	2 (4.4)	17 (25.8)	10 (40.0)	NA	
Microscopic *	8 (27.6)	0 (0.0)	8 (47.1)	6 (60.0)	NA	0.56 **
Macroscopic *	7 (24.1)	1 (50.0)	4 (23.5)	3 (30.0)	NA	
Unspecified *	14 (48.3)	1 (50.0)	5 (29.4)	1 (10.0)	NA	
Negative	73 (50.7)	29 (64.4)	35 (53.0)	9 (36.0)	NA	
Not evaluated	19 (13.2)	6 (13.3)	3 (4.5)	2 (8.0)	8 (100)	
Unknown	23 (16.0)	8 (17.8)	11 (16.7)	4 (16.0)	NA	
Metastasis at diagnosis	6 (4.2)	0 (0.0)	2 (3.0)	3 (12.0)	1 (12.5)	0.06
Bone	2 (1.4)	0 (0.0)	1 (1.5)	1 (4.0)	0 (0.0)	
Brain	1 (0.7)	0 (0.0)	0 (0.0)	1 (4.0)	0 (0.0)	
Lung	1 (0.7)	0 (0.0)	1 (1.52)	0 (0.0)	0 (0.0)	
Other †	2 (1.4)	0 (0.0)	0 (0.0)	1 (4.0)	1 (12.5)	

**CTx**, chemotherapy; **IQR**, interquartile range; **RTx**, radiation therapy; NA, not applicable. *p*-values in bold indicate statistical significance, *p <* 0.05 * Percentage calculated out of positive surgical margins. ** The *p*-value represents the statistical significance of the observed differences in microscopic and macroscopic positive margins. † Other metastasis sites exclude bone, brain, lung, liver, and distant lymph nodes.

**Table 3:** Multivariate cox proportional regression analysis of 5-year mortality in patients treated for pleuropulmonary blastoma.

		aHR	95% CI	*p*-Value
Treatment modality	Surgery	Reference		
	Surgery/CTx	3.20	0.62–16.60	0.20
	Surgery/CTx/RTx	2.38	0.31–18.60	0.40
	CTx	32.30	3.54–294.00	**0.002**
Tumor size	≤5 cm	Reference		
	>5 cm	1.51	0.30–7.49	0.60
Hispanic	No	Reference		
	Yes	2.94	0.96–9.02	0.06
Metastasis at diagnosis	No	Reference		
	Yes	9.14	2.22–37.70	**0.002**
Surgical margins	Negative	Reference		
	Positive	0.41	0.08–2.15	0.30
	Not assessed/unknown	0.40	0.08–1.95	0.30

aHR, unadjusted Hazard Ratio; CI, confidence interval CTx, chemotherapy; RTx, radiation therapy. The *p*-values in bold indicate statistical significance, *p* < 0.05.

## Data Availability

The data presented in this study are available on request from the corresponding author. The data are not publicly available due to specific ethical and privacy considerations.

## Supplementary Materials

The following supporting information can be downloaded at https://www.mdpi.com/article/10.3390/children11040424/s1, Table S1. Bivariate Cox proportional regression analysis of 5-year mortality in patients treated for pleuropulmonary blastoma. Figure S1. Number of patients diagnosed with PPB per year. Table S2. Patient and tumor characteristic comparing patients that presented with metastasis at diagnosis to those that did not. File S2. Post hoc analysis results.
