# Supplementary material for: Patient Characteristics, Treatment, and Survival in Pleuropulmonary Blastoma: An Analysis from the National Cancer Database
Source: Children (Basel). 2024 Apr 2;11(4):424. doi: 10.3390/children11040424 (PMC11049483; doi:10.3390/children11040424)
Supplement: Supplementary file 1 [file children-11-00424-s001.zip › children-2914656-supplementary.pdf]

children

Supplementary Materials

File S1

Table S1. Bivariate Cox proportional regression analysis of 5-year mortality in patients treated for pleuropulmonary blastoma.

Variable		HR	95% CI	*p*-Value
**Age**	(in years)	1.07	0.95–1.21	0.30
**Sex**	Male	Reference		
	Female	1.54	0.71–3.37	0.30
**Race**	White	Reference		
	Black	1.42	0.49–4.12	0.50
	Other	2.26	0.53–9.65	0.30
**Ethnicity**	Non-Hispanic	Reference		
	Hispanic	2.53	1.11–5.78	**0.03**
**Laterality**	Right	Reference		
	Left	1.12	0.47–2.64	0.80
	Both	34.20	3.87–303.00	**0.001**
**Tumor size**	≤5 cm	Reference		
	>5 cm	3.21	0.74–14.00	0.12
**Metastasis at**	Not present	Reference		
**diagnosis**	Present	6.28	2.15–18.30	**<0.001**
**Surgical margins**	Negative	Reference		
	Positive	1.40	0.52–3.77	0.50
	Microscopic	Reference		
	Macroscopic	1.14	0.19–6.80	0.90
	Unspecified	0.57	0.06–5.53	0.60
**Treatment modality**	Surgery	Reference		
	Surgery/CTx	4.98	1.13–21.90	**0.04**
	Surgery/CTx/RTx	4.96	0.96–25.60	0.06
	CTx	28.60	5.75–142.0	**<0.001**
**Years of diagnosis**	2004–2009	Reference		
	2010–2014	0.25	0.06–1.16	0.08
	2015–2019	1.20	0.54–2.68	0.7

p-values in bold indicate statistical significance; CI, confidence interval; CTx, chemotherapy; HR, unadjusted Hazard Ratio; RTx, radiation therapy.

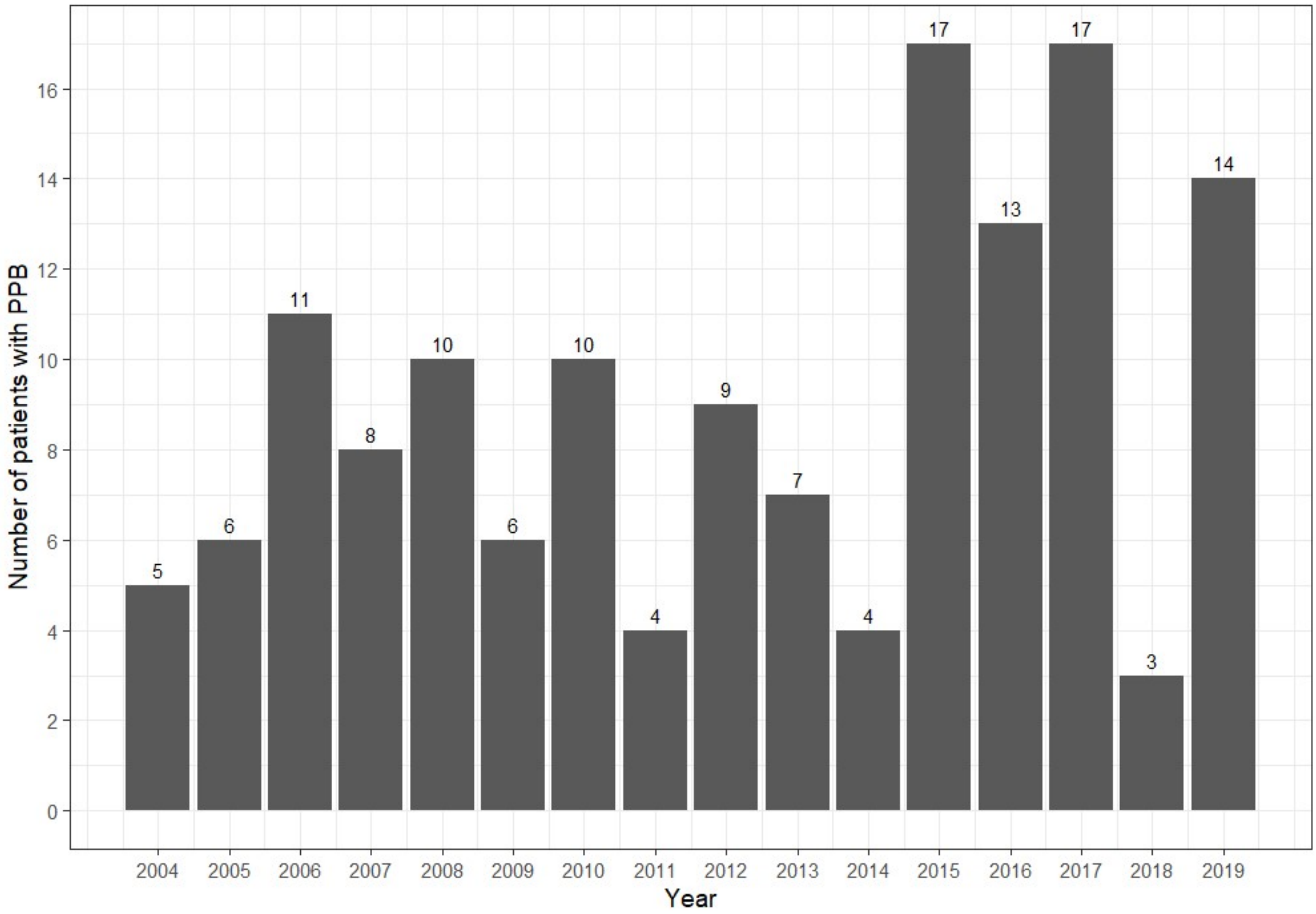

Figure S1. Number of patients diagnosed with PPB per year.

Table S2. Patient and tumor characteristics comparing patients that presented with metastasis at diagnosis to those that did not.

	**Metastasis**		
	**No**<br>**n= 138**	**Yes**<br>**n= 6**	***p*-Value**
**Age, median years [IQR]**	2 [1–3]	2 [1–3]	0.86
**Sex, n (%)**			0.69
Male	64 (46.4)	2 (33.3)	
Female	74 (53.6)	4 (66.7)	
**Race, n (%)**			0.03
White	116 (84.1)	4 (66.7)	
Black	17 (12.3)	0 (0)	
Other	5 (3.6)	2 (33.3)	
**Ethnicity, n (%)**			1
Hispanic	117 (84.8)	5 (83.3)	
Non-Hispanic	21 (15.2)	1 (16.7)	
**Laterality**			1
Right	59 (42.8)	3 (50.0)	
Left	68 (49.2)	3 (50.0)	
Both	1 (0.7)	0 (0.0)	
Unknown	10 (7.3)	0 (0.0)	

**Tumor size (cm), median [IQR]**	81 [47–110]	124 [92–130]	0.93
≤5 cm	27 (19.7)	0 (0.0)	0.21
>5 cm	69 (50.0)	5 (83.3)	
Unknown	42 (30.3)	1 (16.7)	
**Treatment regimens**			**0.04**
Surgery	45 (32.6)	0 (0.0)	
Surgery/CTx	64 (46.4)	2 (33.3)	
Surgery/CTx/RTx	22 (15.9)	3 (50.0)	
CTx	7 (5.1)	1 (16.7)	

File S2

Table S3. Location—upper lobe.

	**Surgery**	**Surgery/CTx**	**Surgery/CTx/RTx**
**Surgery/CTx**	**<0.0001**		
**Surgery/CTx/RTx**	**0.0002**	0.77	
**CTx**	**<0.0001**	**0.002**	**0.001**

Table S4. Location—bronchus/lung unspecified.

	**Surgery**	**Surgery/CTx**	**Surgery/CTx/RTx**
**Surgery/CTx**	**0.04**	-	-
**Surgery/CTx/RTx**	**0.04**	1	-
**CTx**	1	0.09	0.09

Table S5. Positive surgical margins.

	**Surgery**	**Surgery/CTx**
**Surgery/CTx**	**0.002**	-
**Surgery/CTx/RTx**	**<0.0001**	0.06
